# Non-familial cardiomyopathies in Lebanon: exome sequencing results for five idiopathic cases

**DOI:** 10.1186/s12920-019-0478-7

**Published:** 2019-02-14

**Authors:** Marwan M. Refaat, Sylvana Hassanieh, Jad A. Ballout, Patrick Zakka, Mostafa Hotait, Athar Khalil, Fadi Bitar, Mariam Arabi, Samir Arnaout, Hadi Skouri, Antoine Abchee, Bernard Abi-Saleh, Maurice Khoury, Andreas Massouras, Georges Nemer

**Affiliations:** 1 Department of Internal Medicine, Cardiology Division, American University of Beirut Faculty of Medicine and Medical Center (AUBMC), Phase I, 8th floor, Room C-823, PO Box 11-0236, Riad El-Solh, Beirut 1107 2020 Lebanon; 2 Department of Biochemistry and Molecular Genetics, American University of Beirut Faculty of Medicine and Medical Center (AUBMC), Phase I, 8th floor, Room C-823, PO Box 11-0236, Riad El-Solh, Beirut 1107 2020 Lebanon; 3 Department of Pediatrics and Adolescent Medicine, American University of Beirut, Beirut, Lebanon; 4 Saphetor S.A., Lausanne, Switzerland

**Keywords:** Cardiomyopathy, Genetics, Whole exome sequencing, Natriuretic peptide receptor

## Abstract

**Background:**

Cardiomyopathies affect more than 0.5% of the general population. They are associated with high risk of sudden cardiac death, which can result from either heart failure or electrical abnormalities. Although different mechanisms underlie the various types of cardiomyopathies, a principal pathology is common to all and is usually at the level of the cardiac muscle. With a relatively high incidence rate in most countries, and a subsequent major health burden on both the families and governments, cardiomyopathies are gaining more attention by researchers and pharmaceutical companies as well as health government bodies. In Lebanon, there is no official data about the spectrum of the diseases in terms of their respective prevalence, clinical, or genetic profiles.

**Methods:**

We used exome sequencing to unravel the genetic basis of idiopathic cases of cardiomyopathies in Lebanon, a relatively small country with high rates of consanguineous marriages.

**Results:**

Five cases were diagnosed with different forms of cardiomyopathies, and exome sequencing revealed the presence of already documented or novel mutations in known genes in three cases: *LMNA* for an Emery Dreifuss Muscular Dystrophy case, *PKP2* for an arrhythmogenic right ventricle dysplasia case, and *MYPN* for a dilated cardiomyopathy case. Interestingly two brothers with hypertrophic cardiomyopathy have a novel missense variation in *NPR1*, the gene encoding the natriuretic peptides receptor type I, not reported previously to be causing cardiomyopathies.

**Conclusion:**

Our results unravel novel mutations in known genes implicated in cardiomyopathies in Lebanon. Changes in clinical management however, require genetic profiling of a larger cohort of patients.

**Electronic supplementary material:**

The online version of this article (10.1186/s12920-019-0478-7) contains supplementary material, which is available to authorized users.

## Background

The classification and definition of cardiomyopathies has changed significantly over the last 30 years. The substantial advancement in the understanding and diagnosis of the phenotypes associated with the cardiac dysfunction is one reason, but more importantly is the acquired knowledge on the genetic basis of these cardiac disorders. The most recent phenotype-genotype classification by the World Health Federation published in 2013, considers both the morpho-functional phenotype (HCM: hypertrophic cardiomyopathy, DCM: dilated cardiomyopathy, RCM: restrictive cardiomyopathy, ACM (Arrhyhtmogenic Cardiomyopathy), LVNC: left ventricular non-compaction), and the genetic alterations [1]. Genetically, the different forms of the disease are classified under monogenic disorders whereby mutations in a single gene are sufficient to cause a phenotype. Most cases of cardiomyopathy are inherited in an autosomal dominant fashion, with some cases being autosomal recessive, X-linked, or sporadic. So far more than 1000 mutations have been linked to the 5 different classes of cardiomyopathies in more than 40 genes mainly encoding contractile proteins regulating the contraction of the cardiomyocytes [1]. Some genes are specific to one class of cardiomyopathy like *RBM20* in DCM, whereas others like *MYPN* and *TNNI3* are implicated in at least 3 different types of cardiomyopathy [2, 3].

Population based genetic testing and in vitro assessment of defective proteins, have helped tremendously in understanding the molecular basis of the different phenotypes yet, there are many challenges towards implementing common therapeutical and interventional guidelines. Hence, the importance of genetic testing and research in cardiomyopathy for its implications on diagnosis, prognostication, and counseling.

Recent advancements in sequencing technology, and the introduction of Next Generation Sequencing (NGS) allowed for the expansion in knowledge concerning disease-causing genes in patients and families with cardiomyopathy. In Lebanon, there are no previous genetic studies on cardiomyopathies. We are hereby reporting the results of the first exome sequencing done on 6 patients with no family history for the disease.

## Methods

### Patients’ selection

Patients with primary cardiomyopathy (including hypertrophic, dilated, arrhythmogenic, restrictive, and spongiform or left ventricular non-compaction), were identified by their primary cardiologists based on echocardiographic or cardiac magnetic resonance imaging findings, in addition to history, physical examination and other laboratory data. Patients who were found or suspected to have secondary causes for their cardiomyopathy were excluded from the study.

There were no age restrictions for the study; adult as well as pediatric patients were included. The Institution Review Board (IRB) at the American University of Beirut approved the protocol of the study (Protocol Number: IM.MR.01). An written informed consent was obtained from all participants in the study.

### DNA extraction

Blood samples were obtained via venipuncture, and DNA was extracted from peripheral lymphocytes using a DNA extraction kit (Qiagen, Hilden, Germany). The samples were quantified using a NanoDrop (Thermo), at the molecular core facilities at the American University of Beirut, and then stored at − 80 degrees Celsius.

### SureSelect next generation exome sequencing, and sanger sequencing

The samples were prepared per an Agilent SureSelect Target Enrichment kit preparation guide (Santa Clara, United States). Briefly, gDNA was sheared to produce small fragments (150-200 bp). Libraries with sequencer specific adaptors and indices were prepared. Samples were hybridized with biotinylated RNA library baits. Targeted regions were selected using magnetic streptavidin beads. The libraries were amplified and sequenced with Illumina HiSeq 2000/2500 sequencer. Selected variants were verified by Sanger sequencing at the molecular core facility at AUB, as previously described on an ABI3500 platform [4].

### Data analysis

FastQ files were uploaded to the Saphetor platform (https://portal.saphetor.com). The reads were aligned to the UCSC hg19 genome and variants were called using the platform’s consensus method using two variant calling tools. Saphetor’s platform annotates variants by linking them to known disease associations, population statistics, treatment options, clinical trials, scientific literature and a number of pathogenicity metrics (https://saphetor.com/technology_platform/). Each variant is classified by our algorithm using the 5-class score suggested by the ACGS (Association for Clinical Genetic Science) guidelines.

**Fig. 1:**
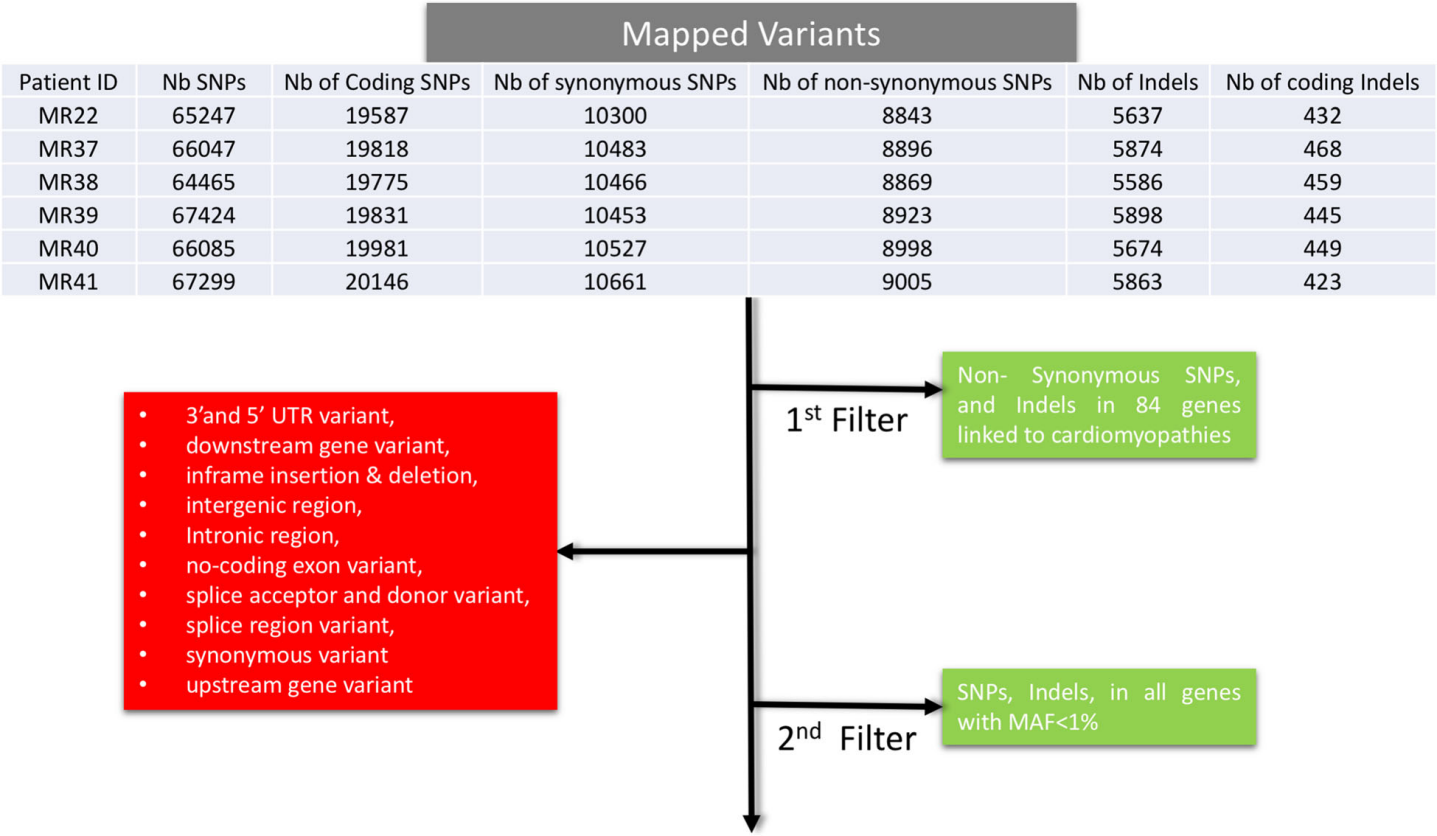
Schematic representation of the data anlysis work flow. The number of variants from each sample is tabulated. The filtered-out variants are highlighted in red, whereas the kept variants followed either path 1 or 2 (green)

The average total number of single nucleotide variants (SNV) and Indels in all samples was around 20,000 and 5500 respectively (Fig. 1). Amongst these, around 8500 SNVs are non-synonymous, and 450 Indels are in the coding regions, and were subject to the first round of filtering against the known 84 genes used for clinical diagnosis of all types of cardiomyopathies (http://www.ambrygen.com/tests/cardionext). All exons in those 84 genes were at least covered 30X. All variants with a minor aller frequency (MAF) in the Gnomad database of less than 1% are included in the first round of filtering against the cardiomyopathy panel. The second round of filtering targeted all variants with frameshift insertion/deletions, or SNVs with predictive deleterious effects as assessed by 13 different in silico criteria (https://saphetor.com/technology_platform /), and with a MAFs< 1%.

## Results

### Patient characteristics

Six eligible patients with different phenotypes were included in the study. The characteristics of patients are presented in Table 1.

**Table 1:** Patient Characteristics and Demographics

Patient	Gender	Age Category	Disease	ICD^a^
MR 37	M	10–20	Arrhythmogenic Right Ventricular Dysplasia	No
MR 38	F	40–50	Emery-Dreifuss Muscular Dystrophy	Yes
MR 39	M	30–40	Hypertrophic Cardiomyopathy	Yes
MR 40	M	40–50	Hypertrophic Cardiomyopathy	No
MR 41	M	40–50	Dilated Cardiomyopathy	No

^a^*ICD* Implantable Cardioverter Defibrillator

### Mutations in known genes

Patient MR38 had been diagnosed in early adulthood with a progressive muscular dystrophy with distribution of muscle weakness and contractures suggestive of Emery-Dreifuss Muscular Dystrophy. At the age of 27 she was found to have non-sustained ventricular tachycardia with grade II diastolic dysfunction, and after a few years she developed symptoms of heart failure. The patient had an implantable cardioverter defibrillator. In this patient, only one variant was classified as pathogenic, and 5 were variants of uncertain significance after the first round of filtering (Table 2). The pathogenic variant in the Lamin A/C gene *LMNA* was previously reported as de novo mutation arising in a family from the United Kingdom (UK). The c.1357C > T (NM_170707.3) variant in exon 7 was detected on one allele and leads to a p.R453W missense mutation predicted to be deleterious.

**Table 2:** Variants filtered based on a MAF < 1% in Exomes and Genomes in 84 known genes associated with cardiomyopathy for patient MR38

Variant	Chromosome	RS ID	Type	Zygosity	Genes	Phenotypes	Function	Coding impact	ClinVar class	Exome Frequency	1000Genomes	Coverage
chr10:69959174 C ⇒ T	chr10	rs71534278	SNV	Heterozygous	MYPN		non-coding exon,coding	missense	Not provided, NA,Pathogenic,Likely Benign	0.0030344	0.00259585	31
chr2:179433611 G ⇒ A	chr2		SNV	Heterozygous	MIR548N,TTN,TTN-AS1		coding,intronic	nonsense				227
chr6:112460443 C ⇒ T	chr6	rs782592337	SNV	Heterozygous	LAMA4		coding	missense		8.23764E-06		137
chr2:179659806 G ⇒ A	chr2	rs199590524	SNV	Heterozygous	TTN		coding	missense		4.94438E-05		123

Patient MR37 was diagnosed with arrhythmogenic right ventricle dysplasia with no family history. The first phase filtering strategy identifies 4 variants, one of which predicted pathogenic, and three with uncertain significance (Table 3). The pathogenic variant is a deletion of one nucleotide on one allele in the gene encoding plakophilin-2 (PKP2). The chr12:32945646 delT variant was first found in one individual of Western European origin, and leads to a frameshift in the readout of the protein with an extended C-terminal domain p.V837 fs930. Filtering for indels and nonsense variants with a MAF < 1% showed one nonsense variant in the *TMEM106C* gene, and deletions in *OR4S1*, *OR2T4*, and *TYRO3* (Additional file 1: Table S1). Probabilities of loss of intolerance (pLI) for these genes didn’t however support a role for them in the underscored phenotype.

**Table 3:** Variants filtered based on a MAF < 1% in Exomes and Genomes in 84 known genes associated with cardiomyopathy for patient MR37

Variant	Chromosome	RS ID	Type	Zygosity	Genes	Phenotypes	Function	Coding impact	ClinVar class	Exome Frequency	1000Genomes	Coverage
chr12:32945646 delT	chr12	rs727504432	Deletion (1)	Heterozygous	PKP2	Cardiomyopathy	coding	frameshift	Likely Pathogenic, Pathogenic			152
chr15:73617434 C ⇒ T	chr15	rs201319883	SNV	Heterozygous	HCN4		coding	missense	Uncertain significance	8.23655E-06		140
chr12:33049482 G ⇒ T	chr12	rs199601548	SNV	Heterozygous	PKP2	Cardiomyopathy	coding	missense	Uncertain significance	0.000167926		9
chr6:7576619 G ⇒ A	chr6	rs142494121	SNV	Heterozygous	DSP	Cardiomyopathy	coding	missense	Uncertain significance, Likely Benign	0.0011205		189

Patient MR41 passed away a couple of months after being recruited at 56 years of age. He was diagnosed with dilated cardiomyopathy alongside his brother who refused to participate in the study. The first filter yielded 81 variants, with only one predicted pathogenic, and 3 with uncertain significance (Table 4). The pathogenic variant on chr10:69959174 C > T affects one allele of the gene encoding Myopalladin (*MYPN*), leading to a missense mutation p.P1112L, previously reported in patients with HCM or DCM (Kimura, 2016 #5)(Duboscq-Bidot, 2008 #18). Two of the three variants with uncertain significance lead to missense mutations in the genes encoding *LAMA4*, *TTN*, and the third affects the region harboring *miR548N*, *TTN*, and *TTN-AS1* (Table 4).

**Table 4:** Variants filtered based on a MAF < 1% in Exomes and Genomes in 84 known genes associated with cardiomyopathy for patient MR41

Variant	Chromosome	RS ID	Type	Zygosity	Genes	Phenotypes	Function	Coding impact	ClinVar class	Exome Frequency	1000Genomes	Coverage
chr10:69959174 C ⇒ T	chr10	rs71534278	SNV	Heterozygous	MYPN		non-coding exon,coding	missense	Notprovided,NA,Pathogenic,Likely Benign	0.0030344	0.00259585	31
chr2:179433611 G ⇒ A	chr2		SNV	Heterozygous	MIR548N,TTN,TTN-AS1		coding,intronic	nonsense				227
chr6:112460443 C ⇒ T	chr6	rs782592337	SNV	Heterozygous	LAMA4		coding	missense		8.23764E-06		137
chr2:179659806 G ⇒ A	chr2	rs199590524	SNV	Heterozygous	TTN		coding	missense		4.94438E-05		123

### Mutation in a novel gene

Finally, two brothers with hypertrophic cardiomyopathy MR39 and MR40 were screened for potential recessive variants given the absence of a family history link. Results from the first round of filtering failed to show any common variant between the two siblings in the common known genes involved in cardiomyopathies (Tables 5, and 6). The second filtering phase yielded amongst others 20 homozygous variants, and only one shared by the two siblings with an overall MAF of 0.04% (Additional file 1: Tables S2 and S3). The chr1:153659131G > A variant leads to a missense variant p.V590 M that could hamper the function of the natriuretic receptor NPR1. However, since the variant is present on one allele in MR40 and on both in MR39, and since there are no DNA samples from the parents, we compiled all heterozygous variants that are only shared between the two siblings, with a MAF < 1%, and that are not found in 200 exomes from Lebanese origin. The data summarized in Additional file 1: Table S4, showed 73 missense variants with some genes being directly or indirectly deregulated in hypertrophy like Fibulin2, and Fibrillin 3.

**Table 5:** Variants filtered based on a MAF < 1% in Exomes and Genomes in 84 known genes associated with cardiomyopathy for patient MR39

Variant	Chromosome	RS ID	Type	Zygosity	Genes	Phenotypes	Function	Coding impact	ClinVar class	Exome Frequency	1000Genomes	Coverage
chr2:220283400 C ⇒ A	chr2	rs375719734	SNV	Heterozygous	DES		coding	missense	Uncertain significance	0.0001205		45
chr22:19754227 G ⇒ C	chr22	rs755937050	SNV	Heterozygous	TBX1		intronic,coding	missense		0.0005598		38
chr14:76447058 G ⇒ A	chr14	rs4252315	SNV	Heterozygous	TGFB3		coding	missense	Likely Benign	0.0002059	0.00219649	110
chr4:114290816 G ⇒ C	chr4	rs79577190	SNV	Heterozygous	ANK2		coding	missense	Likely Benign	0.0006609	0.00159744	66
chr14:23886409 G ⇒ C	chr14	rs3729823	SNV	Heterozygous	MHRT,MYH7		coding,intronic	missense	NA,Benign	0.0074622	0.00519169	135

**Table 6:** Variants filtered based on a MAF < 1% in Exomes and Genomes in 84 known genes associated with cardiomyopathy for patient MR40

Variant	Chromosome	RS ID	Type	Zygosity	Genes	Function	Coding impact	ClinVar class	Exome Frequency	1000Genomes	Coverage
chr2:179434186 T ⇒ A	chr2	rs201095164	SNV	Heterozygous	MIR548N,TTN,TTN-AS1	coding,intronic	missense	Uncertain significance	0.0001499		134
chr2:179403853 T ⇒ C	chr2	rs200544701	SNV	Heterozygous	MIR548N,TTN,TTN-AS1	coding,intronic,non-coding exon	missense	Uncertain significance	0.00015744		155
chr19:35530580 G ⇒ A	chr19	rs150721582	SNV	Heterozygous	SCN1B	coding	missense	Uncertain significance, Likely Benign	0.00049492	0.000399361	34
chr22:19754227 G ⇒ C	chr22	rs755937050	SNV	Heterozygous	TBX1	intronic,coding	missense		0.0005598		16
chr10:88446830 G ⇒ A	chr10	rs121908338	SNV	Heterozygous	LDB3	intronic,coding,splicing	missense	Uncertain significance, Benign,Likely Benign	0.00455428	0.00778754	28
chr14:76447058 G ⇒ A	chr14	rs4252315	SNV	Heterozygous	TGFB3	coding	missense	Likely Benign	0.00020591	0.00219649	86
chr4:114290816 G ⇒ C	chr4	rs79577190	SNV	Heterozygous	ANK2	coding	missense	Likely Benign	0.00066088	0.00159744	51
chr14:23886409 G ⇒ C	chr14	rs3729823	SNV	Heterozygous	MHRT,MYH7	coding,intronic	missense	NA,Benign	0.00746219	0.00519169	101

## Discussion

This is the first study in Lebanon and in the region where whole exome sequencing technology has been implemented on patients with primary cardiomyopathy without a previous family history of the disease.

Cardiomyopathies are a heterogeneous group of disorders with underlying genetic alterations that contribute to disease generation and progression. Mutations in sarcomeric genes play a major role in the pathogenesis of cardiomyopathy, particularly HCM and DCM, with 40–50% of patient with HCM carrying mutations in a sarcomeric gene [5]. These mutations can affect both contractile (myosin, actin), and non-contractile proteins (titin, z-disc proteins) of the sarcomere. Most of the mutations have an autosomal dominant or X-linked inheritance.

There are 2 mechanisms of disease generation in single nucleotide variants, and these are missense single nucleotide variants (characteristic of *MYH7* variants), and nonsense variants (characteristic of *MYBPC3* mutations) [5]. There exists a high degree of overlap and interaction between the different causative genes, and the different cardiomyopathy phenotypes. The same gene can be implicated in more than one phenotype. For example, the *MYH7* gene is implicated in HCM, DCM, RCM, and LVNC [5–7]. The proposed explanation for this observation is that different variants in the same gene lead to different functional alterations in that gene, and eventually manifest as a particular phenotype [7]. Moreover, it becomes even more complex when the phenomenon of double heterozygosity is taken into consideration, pointing out to the extensive interactions between the different gene products. For example a study on 41 patients with DCM belonging to an extended Italian family, revealed that family members with a severe form of the disease requiring early heart transplant were doubly heterozygous for *LMNA* (*LMNA*:c.656A > C) and *TTN* (*TTN*:c.14563C > T), while the other family members with the less severe form were not [8].

Exome sequencing has been used for several years to unveil the underlying genetic mechanisms of disease, particularly in the field of cardiomyopathy where several novel disease-causing mutations have been described using this technique [9, 10]. In addition, the development of extensive genetic databases along with the use of WES technology, has allowed investigators to identify novel genes that may be involved in cardiomyopathy, which would therefore help explain cases without a clear underlying genetic component. One example is a study done by Xu et al. where they identified 10 putative genes associated with HCM using WES and several software programs including Transmission and De Novo Association (TADA) program, and the ToppGene program. In addition a protein-protein interaction network was formed to illustrate the interaction and cross talk between the different gene products [11]. Thus, WES is a powerful diagnostic tool. WES has an increased ability to detect a disease causing pathogenic variant compared to commercially available genetic panels. In one study the detection rate by WES was 8% higher when compared to commercial panels (26.5% vs. 18%, *p* = 0.04) [12] .

Our patient MR38 was initially diagnosed with a progressive muscular dystrophy, but without an exact diagnosis of the specific type of dystrophy. Emery Dreifuss Muscular Dystrophy (EDMD) at that time was contemplated, however due to the unavailability of genetic testing, the diagnosis could not be confirmed. The detected mutation in *LMNA* gene allowed us to confirm the diagnosis of EDMD [13]. Many mutations in *LMNA* have been described so far (464 different mutations in 2251 subjects) (http://www.umd.be/LMNA/), however the mutation that we detected in our patient (c. 1357C > T) is the most common mutation reported in patients with EDMD present in around 12% of patients [14] .

Patient MR37 diagnosed with ARVD was found to have a damaging mutation in *PKP2* gene. ARVD is a condition characterized by cardiomyocyte loss, and fibrofatty replacement thus predisposing to ventricular arrhythmias and sudden death. The incidence of ARVD is estimated to be between 1:5000 and 1:2000, and in many cases without a familial pattern of inheritance [15]. *PKP2* is one of the most commonly mutated genes in patients with ARVD. *PKP2* gene encodes the protein plakophilin-2, which in association with other proteins including plakoglobin and desmoplakin, contributes to the structural and functional integrity of cardiac desmosomes [15–17]. Therefore, a mutation in *PKP2* is expected to result in impaired cell-cell mechanical and electrical connection. Despite the fact that Tintelen et al. demonstrated that only familial ARVD is related to mutations in *PKP2* [16]*,* our patient did not have a family history of ARVD. One possible explanation for this observation is the incomplete penetrance and variable expressivity of *PKP2* mutations described by Dalal et al. [18]. However, without access to genetic testing of the patient’s family members, we cannot confirm that the mutation detected in this patient is actually a de novo mutation.

Patient MR41 with DCM was found to have a missense mutation in *MYPN* gene. DCM is a common cause of heart failure and heart transplantation. The estimated prevalence is 36.5 per 100,000. Familial forms of DCM may be associated with other cardiac and skeletal manifestations. Myopalladin is an important structural protein present at the Z and I lines of striated muscle. Through its central and C-terminal domains, MYPN binds to alpha-actinin and nebulette, respectively, allowing actin and titin to attach to the Z-disc. On the other hand, via its N-terminal domain, it interacts with cardiac ankyrin repeat protein (CARP), and therefore plays a role in muscle gene expression [19, 20]. The mutation that was found in our patient (p.1112 L) has been described and studied recently by Duboscq-Bidot et al. The study showed that when transiently overexpressed into rat neonate cardiomyocyte (RNC), the mutated protein resulted in drastic reduction in cell survival over time [19]. This therefore points out to the importance of a structurally and functionally intact Myopalladin protein on cardiomyocyte structure and function. However these results do not explain the association of Myopalladin mutations with various types of cardiomyopathy, including DCM, HCM, and RCM [6]. A study by Purevjav et al. demonstrated that mutations in different regions of MYPN result in different disruptions in protein-protein interactions, and finally culminate in different clinical syndromes. In this study, the mutation affecting the central and C-terminal domains of MYPN (p.Q529X) resulted in impaired recruitment of alpha-actinin, desmin, and CARP finally leading to RCM. On the other hand the mutation affecting the N-terminal domain (p.Y20C) resulted in impaired expression of CARP, alpha-actinin, and NEBL leading to HCM or DCM [20].

The 2 brothers MR39 and MR40 had HCM with no other family members affected with the disease. Genetic sequencing did not show any mutations in genes known to be causative of HCM. However, the mutation in the natriuretic peptide receptor 1 (NPR1) could possibly explain the phenotype, or at least could have contributed to it. *NPR1* gene codes for natriuretic peptide receptor (NPR), a membrane guanylyl cyclase which has an extracellular ligand-binding domain, a transmembrane domain, and an intracellular catalytic domain. It functions as a receptor for both atrial natriuretic peptides type A and B (NPPA and NNPB). Studies on animal models have shown that mice lacking *Npr1* have extensive cardiac hypertrophy disproportionate to the degree of associated hypertension, with elevated levels of NPPA and NPPB in regions with ventricular hypertrophy and fibrosis, in addition to increased expression of cytoskeletal elements. Thus, there is a possible role of NPPA and NPPB in preventing abnormal cardiac hypertrophy [21, 22]. In addition, the knockout mice’s hearts showed initially impaired relaxation and eventually developed impaired contractility. Interestingly our two patients have elevated systolic pressure (160 mmHg and 140 mmHg) which can be also related to the malfunctioning of the NPR1 receptor. Since there is not sufficient evidence to confirm that *NPR1* mutations can cause or contribute to HCM, further genetic studies, particularly WES should be performed in patients with HCM, especially the 40% without a clearly diagnosed underlying genetic cause of the disease.

## Conclusion

The results of the present study argue for a much-needed registry for patients with cadiomyopathies in Lebanon, that would lead to a better therapeutic approach and guidelines in light of reoccurrence of founder mutations like the one our group did highlighten in pediatric dilated carnitine related cardiomyopathy [23, 24].

## Additional files

Additional file 1: **Table S1.** Whole-exome sequencing filtered results of patient MR37 with non-sense variants or Indels minor allele frequencies’ < 1%. **Table S2.** Whole-exome sequencing filtered results of patient MR39 with homozygous SNPs or Indels minor allele frequencies’ < 1%. **Table S3.** Whole-exome sequencing filtered results of patient MR40 with SNPs or Indels minor allele frequencies’ < 1%. **Table S4.** Shared variants between MR39 and MR40 with MAF < 1%. (DOCX 369 kb)

## Abbreviations

ARVD: Arrhythmogenic Right Ventricular Dysplasia
DCM: Dilated Cardiomyopathy
EDMD: Emery Dreifuss Muscular Dystrophy
HCM: Hypertrophic Cardiomyopathy
LVNC: Left Ventricular Non-Compaction
MAF: Minimal Allele Frequency
NGS: Next Generation Sequencing
RCM: Restrictive Cardiomyopathy
SNV: Single Nucleotide Variant
