# Supplementary material for: Non-familial cardiomyopathies in Lebanon: exome sequencing results for five idiopathic cases
Source: BMC Med Genomics. 2019 Feb 14;12:33. doi: 10.1186/s12920-019-0478-7 (PMC6375196; doi:10.1186/s12920-019-0478-7)
Supplement: Supplementary file 1 — Table S1. Whole-exome sequencing filtered results of patient MR37 with non-sense variants or Indels minor allele frequencies’ < 1%. Table S2. Whole-exome sequencing filtered results of patient MR39 with homozygous SNPs or Indels minor allele frequencies’ < 1%. Table S3. Whole-exome sequencing filtered results of patient MR40 with SNPs or Indels minor allele frequencies’ < 1%. Table S4. Shared variants between MR39 and MR40 with MAF < 1%. (DOCX 369 kb) [file 12920_2019_478_MOESM1_ESM.docx]

**Table S1.** Whole-exome sequencing filtered results of patient MR37 with non-sense variants or Indels minor allele frequencies’ <1%

**Table S2.** Whole-exome sequencing filtered results of patient MR39 with homozygous SNPs or Indels minor allele frequencies’ <1%

**Table S3.** Whole-exome sequencing filtered results of patient MR40 with SNPs or Indels minor allele frequencies’ <1%

**Table S4.** Shared variants between MR39 and MR40 with MAF<1%
